# Sir George Newman's Report: The Year's Work in School Medical Inspection

**Published:** 1914-01-31

**Authors:** 


### SIR GEORGE NEWMAN'S REPORT.

### The Year's Work in School Medical Inspection.

The Annual Report of the Chief Medical Officer of the Board of Education for 1912 is, as usual, very interesting reading. The able compilation of returns from the various quarters, the admirable condensation of information, and the careful tabulation of statistics which it offers make it a valuable reference manual. The first chapter is devoted to a report on the administration of school medical work. It shows how cordially the various local authorities all over England and Wales are girding their loins to accomplish the vital task set them by the Act. Every local authority now has its school doctor, its nurse, and its system of medical inspection of schools. Many have also inspection and treatment centres, and the number of clinics devoted to the treatment of defective school children is rapidly increasing. All this is highly encouraging. So, too, is the fact that school doctors, now that the routine work has become systematised and they have more leisure and opportunity for special work, are interesting themselves in special questions. Sir George publishes a list of the special reports which have been published during the year. These range over a large number of interesting and important subjects, from a comparison between Jew and Gentile school children to an elaborate investigation of the condition of the water in certain swimming baths.

### Medical Inspection of Secondary Schools.

Encouraging progress has been made in regard to the medical inspection of secondary schools. Proprietary and public—really private—schools still lag behind. Some of these receive grants in aid from the Board, and it is high time that they should be subject to routine medical inspection as are the schools which receive State aid. It is gratifying to note that one or two such schools have realised their responsibilities in the matter and instituted medical inspection on their own account. Thus a complete scheme of inspection is in being at Wycombe Abbey School. Endowed and grammar schools are on a somewhat similar footing, inasmuch as they are not under the local authority, which is only concerned in providing that bursary and scholarship pupils are properly inspected. The time has come for a general scheme of inspection of all schools, and it is to be hoped that the Board will take the initiative in the matter and draft legislation to enforce such inspection. Only by such a general scheme can we arrive at a general conclusion regarding the health of the rising generation, and compare the physical fitness of the children of the middle class and upper class with those who are attending schools directly under the control of the local authority.

The second chapter is devoted to a consideration of the physical condition of children attending the elementary schools. The conclusions are stated thus at the end of the Report: "Out of the six million children registered on the books of the elementary schools of England and Wales, about 10 per cent. suffer from serious defect of vision; from 1 to 3 per cent. have suppurating ears; about 10 per cent. have adenoids, inflamed tonsils, or enlarged cervical glands, requiring surgical treatment; about 1 per cent. have ringworm; 1 per cent. suffer from tuberculosis of readily recognisable form; from 1 to 2 per cent. are affected with heart disease; from 30 to 40 per cent. have unclean heads or bodies; and probably more than half the children are in need of dental treatment."

### Tuberculosis and Heart Disease.

Two special chapters are devoted to tuberculosis and heart disease in children. Owing to the lack of standardisation of defects and the want of agreement between inspectors with regard to the interpretation of certain physical signs, the evidence of the statistics given in these chapters cannot be accepted as conclusive. Certainly, so far as heart disease is concerned, it is probable that the percentage of defective children is over rather than under estimated. So, too, with regard to tuberculosis. There is a wide discrepancy between the diagnoses of various inspectors. Some go entirely on the physical signs, some on the physical signs plus the history and general nutrition, others again on signs, symptoms, and the additional information gleaned by special tests; in the majority of cases the diagnosis is in the nature of a guess. All these considerations make it evident that we want some standard by means of which the degree of defectiveness in regard to these two diseases can be determined with some certainty. While that is wanting the statistics must necessarily be inconclusive and unreliable.

An interesting chapter is devoted to treatment. Sir George shows how rapidly the local authority is becoming alive to its responsibilities in this matter. School clinics, voluntary or rate aided, are springing up everywhere; the opposition of the private practitioner has ceased to be a considerable factor against them, for he has come to see that it is in his own interests that the parent is urged to seek early treatment for defects discovered at the routine inspections.

### Hospitals and School Children.

It should be added that a note deals with the use of hospitals in this connection. At present a very large number of school children are still treated at the hospitals. In previous Reports the advantages and difficulties of using the hospitals for the treatment of school children have been discussed at length, and Sir George offers no new remarks under this heading, contenting himself with the conclusion that "it is necessary that this use of hospitals should be carefully watched and properly controlled." Yet this control is, and must of necessity be, inadequate, since the local authority, even if it subsidises the hospital, has no say in the staffing of the institution, and therefore no proper control in the strict sense of the word.

### The Need for Special Clinics.

For this reason it is increasingly necessary that school children should be drafted to special clinics. The reasons against their being treated at public hospitals, except at children's hospitals, are, in our opinion, too important to be overlooked. The school clinic, under proper control, is no rival but an auxiliary to the hospital, and there is no reason whatever that their interests should clash. A chapter is devoted to these clinics. They are now properly grouped into three main classes—the inspection clinic, the treatment clinic, and the dental clinic; of these the last are the most numerous, and are doing excellent work, though there is ample room for a further increase in their numbers.

A most interesting article is that which deals with medical inspection and juvenile employment. Sir George rightly remarks that the school doctor should pay special attention to the economic potentialities of leavers. "Owing to the fact that a very large proportion of employed children, possibly upwards of 50 per cent., are engaged in unskilled work, it is clear that no provision is made for their future needs, capacity, and competency. In order to prevent boys and girls from being spoilt physically, mentally, and even morally, by their too early emergence into the ranks of the employed, and by the insufficient safeguarding and husbanding of their physical powers and resources, it is very desirable that guidance and advice in the choice of occupations suited to their capacity should be available."

### The School Doctor and Employment.

"This is where school hygiene in its largest sense should be brought into direct application to industry. Unless the school doctor is in a position to guide and advise from a physical standpoint in regard to questions of the employment of the child at or about the time of leaving school, he is not fulfilling his whole duty, and is not justifying one of the particular reasons of his existence in the State." These are sound suggestions which every school doctor should earnestly consider. Further interesting chapters in the Report deal with schools for mothers and mothercraft; physical training; and special schools for blind or physically defective children. In all these Sir George considers the case from various points; his conclusions are generally sound, and his information is always interesting.

After reading the Report one is forced to the conclusion that things are moving in the right direction, and that there is much—even amidst this welter of statistics that go to prove the gradual deterioration of the race—for which we can be thankful. It is an optimistic Report in the best sense of the word, for it shows encouraging signs behind that gloomy curtain of defects which overhangs the picture as drawn in the second chapter on the physical condition of children in the elementary schools.

